# Rearranged During Transfection Fusions in Non-Small Cell Lung Cancer

**DOI:** 10.3390/cancers11050620

**Published:** 2019-05-03

**Authors:** Connor O’Leary, Wen Xu, Nick Pavlakis, Derek Richard, Ken O’Byrne

**Affiliations:** 1 Department of Medical Oncology, Princess Alexandra Hospital, Brisbane, QLD 4102, Australia; wen.xu@health.qld.gov.au (W.X.); k.obyrne@qut.edu.au (K.O.); 2 Translational Research Institute, Brisbane, QLD 4102, Australia; derek.richard@qut.edu.au; 3 Royal North Shore Hospital, Sydney, NSW 2065, Australia; nick.pavlakis@sydney.edu.au

**Keywords:** non-small cell lung cancer, rearranged during transfection, RET fusions

## Abstract

Identifying and targeting specific oncogenic drivers has become standard of care in the routine management of patients with lung cancer. Research is ongoing to expand the number of drug targets that can offer clinically meaningful outcomes. Rearranged during transfection (RET) fusions are the latest oncogenic driver alterations that show potential as a drug target. RET fusions occur in 1–2% of non-small cell lung cancer (NSCLC) cases. They are more commonly associated with younger age, female gender, non-smokers and Asian ethnicity. The RET kinase is abnormally activated through fusion with a partner protein such as KIF5B, CCDC6 or NCOA4. This leads to downstream intracellular signalling and enhancement of gene transcription and cell proliferation. The effectiveness of multi-kinase inhibitors in RET positive NSCLC has been explored in early phase and retrospective studies. From these studies, the most effective agents identified include cabozantanib and vandetanib. Overall response rates (ORR) vary from 18–47% across studies. In general, these agents have a manageable toxicity profile, although there are a number of off-target toxicities. Similar to the increased activity in ALK rearranged disease, pemetrexed has demonstrated superior response rates in this patient group and should be considered. Selective RET inhibitors, including LOXO-292 and BLU-667, are progressing in clinical trials. LOXO-292 has demonstrated an impressive ORR of 77% in RET positive solid tumours. It is anticipated this agent will be an effective targeted therapeutic option for patients with RET positive lung cancer.

## 1. Introduction

The therapeutic landscape in the field of lung cancer is continually expanding with an increasing emphasis now placed on a personalised approach to patient care, based on the presence or absence of specific oncogenic drivers/mutations. The discovery of targetable oncogenic driver mutations and fusion proteins including epidermal growth factor receptor (EGFR) exon 19 deletion and exon 21 L858R mutation, anaplastic lymphoma kinase (ALK) translocation and ROS-1 rearrangement along with the development of effective selective small molecules has led to better treatment options, as well as improved patient survival, symptom control and quality of life [1,2,3,4]. Research is ongoing to identify further druggable targets. Rearranged during transfection (RET) mutations/fusions represent one such target with drug development efforts focused on identifying agents that can be used to treat this subgroup of lung cancer patients.

## 2. Pathological and Clinical Characteristics

RET is a transmembrane receptor protein tyrosine kinase present on the surface of a number of tissues types including the nervous system, adrenal medulla and thyroid [5]. Alterations in RET can result in gain or loss of function with RET loss of function resulting in conditions such as Hirschsprung’s disease [6]. Abnormal RET activation occurs by two mechanisms associated with malignancy, mutations and fusions. Both somatic and germline RET mutations have been described and are most commonly found in medullary thyroid carcinoma and in patients with multiple endocrine neoplasia syndromes [7].

**Figure 1:**
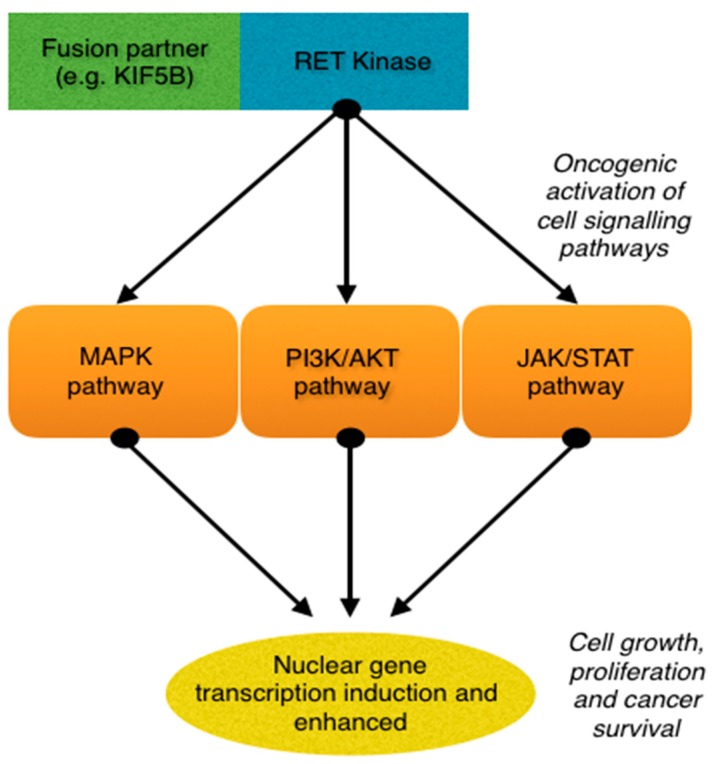
Rearranged during transfection (RET) fusion causing oncogenic activation of downstream intracellular signalling pathways.

RET fusions rather than mutations are typically present in non-small cell lung cancer (NSCLC). A number of RET fusion partners have been described, however the most common variant in NSCLC is the KIF5B fusion partner [8]. KIF5B is reported in 50–70% of RET fusion positive cases of NSCLC. Other fusion partners such as CCDC6 and NCOA4 have been described, but are present less frequently [9,10]. When a fusion partner is present in combination with RET, this results in activation of the oncogenic tyrosine kinase domain of RET [11]. Subsequently, this leads to autophosphorylation of RET and downstream cell signalling through intracellular pathways such as the mitogen-activated protein kinase (MAPK), PI3K/AKT and Janus kinase/signal transducer and activator of transcription (JAK/STAT) pathways (Figure 1). RET fusions behaving in this manner result in oncogenic activation promoting unchecked cellular proliferation [12,13].

RET fusions occur in 1–2% of NSCLC. Like other oncogenic driver mutations in lung cancer, patients with RET fusions are typically associated with younger age, female gender, non-smokers and Asian ethnicity [14,15]. They tend to occur most commonly in patients with lung adenocarcinoma but have also been described in cases of mixed adenosquamous histology [9]. A study of predominantly Caucasian patients noted a difference in clinical patterns compared to other studies. In particular, this study found contrasting higher rates of male patients and smokers with RET fusion alterations in their patient cohort [16]. This may reflect some variability between specific disease characteristics in patients of differing ethnicity.

## 3. Current Research

There is increasing evidence for a variety of agents that have activity against NSCLC tumours that harbour a RET fusion. The majority of data have come from studies of multikinase inhibitors. Selective RET inhibitors have successfully demonstrated impressive disease response rates. These agents are now progressing through clinical trials.

### 3.1. Multikinase RET Inhibitors

A phase 2 study of cabozantanib in 26 patients with RET positive lung cancer showed an overall response rate (ORR) of 28%. The study reported a median progression free survival (PFS) of 5.5 [95% confidence interval (CI), 3.8–8.4] months and overall survival (OS) of 9.9 [95% CI, 8.1-not reached (NR)] months. The majority of toxicity related issues were grade 1 and 2 in severity relating to liver function disturbance, gastrointestinal upset and skin toxicity [17]. Vandetanib was evaluated in a phase 2 study in patients with pretreated RET positive NSCLC. Eighteen patients were enrolled in this study. An ORR of 18%, PFS of 4.5 [95%, CI, not specified (NS)] months and OS of 11.6 [95%, CI, NS] months was observed. Toxicity was acceptable, predominately relating to grade 1 and 2 hypertension, diarrhoea and rash [18]. A Japanese group performed a similar phase 2 study using vandetanib. In this group of 19 patients, an ORR of 47%, PFS of 4.7 [95% CI, 2.8–8.5] and OS of 11.1 months [95% CI, 9.4-NR] was observed, however, higher rates of grade 3 and 4 events were described [19]. Twenty five patients with RET fusion positive NSCLC were treated with lenvatinib. The ORR was 16% and PFS was 7.3 [95% CI, 3.6–10.2] months in this study. Grade 3 treatment related events occurred in 92% of cases [20]. A retrospective assessment of an international registry of American, Asian and European patients assessed the response of 53 RET positive lung cancer patients to various multikinase inhibiting agents. Drugs used included cabozantanib, vandetanib, sunitinib, sorafenib, alectanib, lenvatinib, nintedanib, ponatinib, and regorafenib. The most efficacious drugs in this setting were cabozantanib, vandetanib and sunitinib. Rates of complete or partial response were 37%, 18%, and 22%, respectively. Median PFS was 2.3 months [95% CI, 1.6–5.0] and OS was 6.8 months [95% CI, 3.9–14.3] for the whole group. There was little difference in PFS and OS when stratified by drug, cabozantanib, vandetanib or sunitinib: PFS of 3.6 [95% CI, 1.3–7.0], 2.9 [95% CI, 1.0–6.4] and 2.2 [95% CI, 0.7–5.0] months, respectively, and OS of 4.9 [95% CI, 1.9–14.3], 10.2 [95% CI, 2.4-NR] and 6.8 months [95% CI, 1.1-NR], respectively [21] (Table 1). Preclinical study suggests a rationale for the use of alectinib as a possible therapeutic option in RET positive lung cancer. Kodama et al. found alectinib did not inhibit ROS-1 but instead induced 95% inhibition of RET kinase activity [22]. Alectinib was used to treat four patients with RET positive lung cancer with three patients having received prior RET directed inhibition. Fifty percent of patients experienced a response to treatment including one patient with central nervous system disease [23]. Clinical trials are currently underway evaluating alectanib in this setting.

### 3.2. Selective RET Inhibitors

LOXO-292 is a selective RET kinase inhibitor that has shown activity in the phase 1/2 study LIBRETTO-001, in advanced RET positive solid tumours. This study enrolled 82 patients with advanced solid tumours including 38 patients with RET fusion positive NSCLC. The reported ORR was 77% at the initial assessment with activity described systemically and in the central nervous system. The response was similar irrespective of prior multikinase inhibitor use or fusion partner. Overall LOXO-292 was well tolerated with only two grade 3 events reported. The majority of adverse events were grade 1 and 2 in nature including fatigue, gastrointestinal upset and dry mouth [24]. Due to the marked clinical activity observed, LOXO-292 was granted accelerated approval by the U.S. Food and Drug Administration (FDA). Another selective RET kinase inhibitor, BLU-667, was assessed in a phase 1 study involving RET positive non-small cell lung cancer and thyroid cancer. Fifteen patients with NSCLC were included in the study. ORR was 37% for the whole group at the time of reporting. The majority of drug related toxicity were grade 1 including gastrointestinal upset, liver function derangement and hypertension. Three grade 3 events were reported [25].

### 3.3. Other Strategies

The evaluation of immunotherapy in the context of RET positive NSCLC has been limited. A retrospective review of 74 patients with RET positive NSCLC analysed patient outcomes in this group to immunotherapy. Twelve patients received an immune checkpoint inhibitor. Median duration of response was 1.4 (range, 0.5–8.7) months. Overall survival was unchanged compared to those that did not receive immunotherapy. The median tumour mutational burden (TMB) score was found to be lower than in unselected NSCLC cases. Extent of programmed death ligand 1 (PD-L1) expression, >50%, 1–49% and <1%, did not affect outcomes [26].

Treatment with pemetrexed based regimes (combination or monotherapy) was found to have favourable patient outcomes in RET, ALK and ROS-1 positive NSCLC compared to KRAS mutated NSCLC. Eighteen patients with RET rearranged NSCLC were included. Median PFS was 19 [95% CI, 12-NR] months and ORR was 45%, which was comparable to the ALK and ROS-1 mutate subgroup and significantly improved relative the KRAS mutant subgroup that had a PFS of 6 months (95% CI, 5–9) and ORR 26% [27].

## 4. Conclusions

RET fusions occur infrequently in NSCLC. These alterations tend to be enriched in females, non-smokers and Asian patients but have been identified in a wide spectrum of patient phenotypes. RET fusions result in oncogenic activation of intracellular pathways including MAPK, PI3K/AKT and JAK/STAT leading to unchecked activation of cell proliferation and growth. Inhibition of RET with various multikinase inhibitors has demonstrable activity, however this approach has not yet been adopted or gained regulatory approval. Currently, chemotherapy, in particular pemetrexed containing regimes, or clinical trials of targeted therapy remain favoured options for RET positive lung cancer. Reported responses to immunotherapy have been poor and appear to be similar to other NSCLC groups with other oncogenic drivers, apart from possibly BRAF mutant NSCLC. Improved strategies to target RET with selective RET inhibitors have shown intriguing results in early phase studies. Presently, access to these drugs is only by means of enrolment in clinical studies. With further evaluation, it is likely these agents will be established as a new treatment paradigm for this patient cohort.

## Figures and Tables

**Table 1:** Response rates separated by drugs as reported by each study in RET fusion positive non-small cell lung cancer.

Drug	ORR (%)	PFS (months)	OS (months)
Cabozantanib (17)	28	5.5	9.9
Cabozantanib (21)	37	3.6	4.9
Vandetanib (18)	18	4.5	11.6
Vandetanib (19)	47	4.7	11.1
Vandetanib (21)	18	2.9	10.2
Lenvatinib (20)	16	7.3	NR
Sunitinib (21)	22	2.2	6.8

ORR: objective response rate; PFS: progression free survival; OS: overall survival; NR: not reported.
